# Pilot outcomes and exploration of treatment mechanisms using a culturally adapted version of the unified protocol for transdiagnostic treatment of emotional disorders to improve mental health symptoms, alcohol misuse, functional outcomes, and sleep quality in emergency responders

**DOI:** 10.3389/frhs.2025.1452976

**Published:** 2025-06-16

**Authors:** Eric C. Meyer, Sheila G. Roth, Elizabeth Coe, Daniel J. Taylor, Suzy B. Gulliver

**Affiliations:** ^1^ Department of Counseling and Behavioral Health, University of Pittsburgh, Pittsburgh, PA, United States; ^2^ Department of Social Work, Carlow University, Pittsburgh, PA, United States; ^3^ City of Pittsburgh Department of Public Safety, Pittsburgh, PA, United States; ^4^ Warriors Research Institute, Baylor Scott & White Health, Waco, TX, United States; ^5^ Department of Psychology, University of Arizona, Tucson, AZ, United States; ^6^ Department of Psychiatry, Baylor College of Medicine, Houston, TX, United States

**Keywords:** paramedic, fire fighter, police, first responder, PTSD, quality of life, functional impairment, alcohol

## Abstract

**Introduction:**

Emergency responders encounter frequent trauma and myriad occupational hazards, contributing to concerning rates of posttraumatic stress disorder (PTSD) and related mental health symptoms. These symptoms are each strongly linked with neuroticism/negative emotionality (NNE). Thus, an emotion-focused, transdiagnostic, skills-based treatment approach seems to be a strong match for this population. We sought to address barriers to mental health treatment for emergency responders, including stigma, logistical barriers, and lack of provider knowledge regarding emergency response culture by delivering treatment via telehealth by providers trained in emergency response culture.

**Methods:**

In an uncontrolled pilot trial, we delivered the Unified Protocol for Transdiagnostic Treatment of Emotional Disorders to 30 emergency medical service, police, and fire service personnel.

**Results:**

The large majority (80.0%) completed treatment. Working Alliance Inventory scores were high. Large improvements occurred at post-treatment and one-month follow-up in PTSD symptom severity (Hedges' *g* = 1.1 at post-treatment; *g* = 1.3 at follow-up), depression (*g* = 1.3; 1.3), anxiety (*g* = 1.1; 1.0), functional impairment (*g* = 1.2; 1.1), and quality of life (*g* = .89; .81). Small-to-medium sized improvements occurred in sleep quality (*g* = .42; .69) and engagement in values-consistent behavior (*g* = .34; .77). There were large, theory-consistent improvements during treatment in NNE (*g* = 1.1), difficulties in emotion regulation (*g* = .94), and experiential avoidance (*g* = 1.1), and large associations between changes in these mechanistic variables and improved treatment outcomes.

**Discussion:**

We summarize our cultural adaptation process aimed at maximizing fit of the UP with emergency responders and recommend additional, controlled research examining the UP in trauma exposed populations.

**Clinical Trial Registration:**

NCT05357586.

## 1 Introduction

Emergency response professionals risk their health and well-being to protect the public. Broadly speaking, emergency responders appear to experience elevated risk for developing mental health disorders compared to the general population (1). These elevated risks in emergency responders are linked with myriad occupational stressors (2) including frequent exposure to psychological trauma (3–5) and shift work that interferes with their sleep (6). Level of trauma exposure is associated with suicidal ideation and attempts in emergency responders (7). Moreover, emergency responders were disproportionately impacted by the COVID-19 pandemic and, in turn, experienced an increase in mental health challenges during the pandemic (8–10). While rates vary substantially across studies and disorder categories, estimates of up to one-third of emergency responders develop posttraumatic stress disorder [PTSD; (1, 5, 11–13)]. For the sake of comparison, the estimated lifetime prevalence of posttraumatic stress disorder among people who have been exposed to one or more traumatic event in the general population is 14.5% (14). Other studies report that approximately one-third of emergency responders meet criteria for other common mental health disorders such as depression, anxiety, and sleep disorders (1, 5, 15–18). Published estimates indicate that between one-third and two-thirds of emergency responders report engaging in hazardous alcohol use, which is associated with each of these other mental health challenges and with sleep problems in this population (19–21). Stressors underlying these problems are also linked with elevated rates of occupational burnout, physical health problems such as cardiovascular disease, suicide risk, and greater all-cause mortality, underscoring the need to address the mental health challenges faced by these crucial public safety workers in a holistic manner (2, 7, 22–24). Given that many of the symptoms of these mental health challenges co-occur and overlap across diagnostic categories, treatment for this population should be transdiagnostic in nature (25, 26).

The Unified Protocol for Transdiagnostic Treatment of Emotional Disorders [UP (25, 27, 28);] is a highly structured, empirically supported treatment approach that appears to be a strong match for addressing mental health challenges in emergency responders (26). On a mechanistic level, targeting the negative emotional experiences that underlie and cut across these problems would seem an effective, empirically informed strategy (25, 29). In contrast to disorder-specific treatments that address a single emotional disorder (e.g., depression), the UP engages several evidence-based treatment processes—including mindfulness, cognitive flexibility, and behavioral exposures (30)—and applies them in an integrative manner to address symptoms across diagnostic categories. A growing evidence base supports the utility of the UP for improving emotional and emotion-adjacent symptoms, including anxiety, depression, anger, substance misuse, self-injury, somatization, obsessive-compulsive symptoms, and trauma-related symptoms (26, 31–37). In a meta-analysis of 19 randomized trials (32), there was a medium effect of the UP on pooled anxiety and depression symptoms from pre- to post-treatment (between-groups Hedges' *g* = .45). Moreover, in this study, findings were substantially stronger at follow-up in the 12 RCTs for which follow-up data were available (between-groups *g* = 1.13). To date, four pilot studies have examined the UP as a treatment for PTSD, with all four demonstrating that the UP was associated with reductions in PTSD and other mental health symptoms (26, 34, 35, 37). No published RCTs have tested the UP for PTSD to date. One area where there has been virtually no research is on the effect of the UP on sleep problems even though sleep problems are part of the diagnostic criteria for several categories of emotional disorders (38). One case study and one single case experimental design study both reported improvements in sleep onset latency and sleep quality (39, 40), which the latter study also reported concomitant improvements in emotion regulation and negative emotionality/neuroticism (NNE).

On a mechanistic level, the UP was designed to reduce mental health symptoms by targeting negative emotions, including by teaching skills for more effectively responding to and regulating negative emotions (41, 42). Neuroticism, alternately referred to as negative emotionality (NNE), is the core treatment target in the UP. It is one of the Big 5 personality factors that characterizes the frequency and intensity with which people experience emotions that are typically characterized as negative or unwanted [e.g., fear, anger, sadness, guilt (43);]. NNE is linked with multiple forms of maladaptive coping, including inaction, avoidance, and impulsivity (42, 44–46). In a prospective, observational cohort study of fire fighter recruits who *did not meet criteria for any mental health disorder* upon study entry, higher pre-employment levels of emotionality prospectively predicted (i.e., main effect) and interacted with (i.e., moderated) greater levels of trauma exposure to predict the development of higher levels of PTSD symptom severity over the first three years in fire service (47). Negative alterations in cognition and mood are core features of PTSD, depression, and anxiety (38, 48). NNE is prospectively linked with such a broad range of negative physical and mental health outcomes, including harmful alcohol use, sleep problems, suicide risk, and others that it has been characterized as among the most important risk factors in public health (49–52). Thus, while it is unknown whether emergency response work

Another key factor targeted by the UP is emotion regulation. Extensive research links difficulties in emotion regulation with greater occurrence of negative emotions (53) and symptoms of PTSD, depression, anxiety, and substance use disorders (54–56). We found that fire fighters frequently engage in maladaptive forms of emotion regulation including self-blame and social isolation, which were linked with greater mental health symptoms, particularly when several maladaptive emotion regulation strategies were used in combination (5). In subsequent studies with fire fighters, difficulties in emotion regulation accounted for the link between depression and insomnia and were linked with PTSD symptom severity, sleep difficulties, and alcohol use (16, 17, 57–59). Difficulties in emotion regulation mediated the influence of PTSD symptom severity on relationship satisfaction in fire fighters (60). Conversely, adaptive emotion regulation skills were linked with lower anxiety and depression symptoms in fire fighters (61). Prior research indicates that the UP bolsters adaptive emotion regulation skills and reduces reliance on maladaptive strategies for managing negative emotions (36). The UP also focuses on reducing experiential avoidance, which refers to attempts to avoid, control, or escape from unwanted internal experiences (i.e., cognitions, emotions, physiological sensations), even when doing so interferes with engaging in behaviors that are consistent with one's values and goals (62, 63). The strong associations between experiential avoidance and NNE, along with the negative impact of experiential avoidance on mental health symptoms have been extensively documented (63–68). Several studies have documented decreases in experiential avoidance in diverse client populations receiving the UP (69, 70).

Beyond selecting a treatment approach that addresses the symptoms most commonly experienced by emergency responders, delivering mental health care to this population requires addressing several challenges related to treatment acceptability and feasibility. Emergency responders report stigma and privacy concerns regarding seeking care for mental health symptoms related, at least in part, to occupational stress (16, 71–74). In one study of fire fighters, lack of provider awareness of emergency response culture (53%), fear of breach in confidentiality (51%), and stigma (43%) were rated as the top three barriers to behavioral health care (72). Next, it would be difficult for any clinician to be sufficiently trained in empirically supported treatments for all of the mental health problems commonly found in fire fighters and even less likely that fire fighters would agree to complete each of those protocols sequentially. The UP has the advantage of being transdiagnostic and thus more easily disseminated to and implemented by clinicians who treat fire service personnel. Emergency responders' work schedules often present a challenge related to accessing mental health care (16). Based on our experience working with emergency responders, offering treatment via video conferencing enhances the sense of privacy for many and enhances scheduling convenience, which is important for shift workers. Next, given high levels of trauma exposure in this population, treatment should be trauma informed. Providing treatment that is highly structured and predictable is a core element of trauma informed care. Emergency responders make regular use of manuals and protocols as part of their work; thus, protocol driven treatment may be one way of enhancing predictability and comfort when delivering trauma focused care to this population. Finally, based on our familiarity with emergency response culture, we reasoned that treatment that is active, collaborative, and that emphasizes skill building is a strong fit with emergency response culture.

With these principles in mind, we conducted the first study of the UP in emergency responders, a pilot trial of fire fighters with different combinations of emotional symptoms (26). We began by enlisting the expertise of fire service collaborators in a cultural adaptation process to ensure strong fit between certain elements included in the UP and emergency response culture. Treatment was then delivered via video conferencing by clinicians who were educated in fire service culture. A high proportion (80.3%) of participants completed treatment, underscoring feasibility and acceptability of this highly structured, skills based intervention in this population. We reported medium and large effect size improvements in symptoms of PTSD, depression, anxiety, AUD, and quality of life. Each of these findings was sustained at 1-month follow-up. Thus, receiving the UP was associated with improvements in the types of mental health symptoms most commonly experienced by emergency responders. Moreover, the UP's manualized, skills-based approach seemed to promote treatment acceptability and to be a strong match with emergency response culture.

In the present study, we conducted an uncontrolled pilot trial examining the UP, culturally adapted for use with emergency responders, in a mixed sample of emergency responders. The sample was comprised of emergency medical service professionals (emergency medical technicians and paramedics), fire fighters, law enforcement officers, and emergency room medical personnel. Treatment was delivered individually, either in person or via video conferencing, based on participants' choice in order to maximize access and acceptability. We examined whether emergency responders who received the UP would report improvement in PTSD, depression, anxiety, AUD symptom severity, sleep quality, functional impairment, quality of life, and the extent to which they reported that they were able to consistently engage in behaviors that were consistent with their personal values. We examined treatment outcomes at post-treatment and at 1-month follow-up. Given high rates of trauma exposure in this population and limited data on the utility of the UP for treating posttraumatic stress, we also examined treatment outcomes in the sub-sample of participants who screened positive for PTSD at pre-treatment. We examined the associations between changes in the treatment outcomes and changes in putative treatment mechanisms (NNE, difficulties in emotion regulation, psychological inflexibility). For comparison, we also examined the association between therapeutic working alliance and changes in treatment outcomes. This study expands on our prior study of the UP in fire fighters (26) by examining a mixed sample of emergency responders, examining additional treatment outcomes (functional impairment, sleep quality, values-consistent actions), and exploring the influence of treatment mechanisms. It is the first study to compare the influence of several mechanisms theorized to be targeted in the UP within a trauma exposed sample.

## 2 Material and methods

### 2.1 Institutional review board

The University of Pittsburgh IRB approved all procedures (Study 21120152).

### 2.2 Design

In this uncontrolled pilot trial, all eligible participants received treatment using the UP.

### 2.3 Participants

Male and female, English-speaking emergency response professionals 18 years of age or older residing in Pennsylvania were eligible to participate. Emergency responders were defined as fire fighters, law enforcement officers, emergency medical service personnel, emergency room medical providers, and emergency dispatchers. Potential participants were excluded if they: (1) were unable or unwilling to complete the study procedures; (2) screened positive for a psychotic or bipolar disorder; (3) were experiencing a current suicidal or homicidal crisis warranting immediate intervention at the time of eligibility screening. Potential participants could participate later once stabilized; (4) evidenced severe organic brain impairment as evidenced by a major apparent disruption of consciousness, cognition, speech, or behavior that would likely interfere with treatment; (5) were awaiting the outcome of litigation involving their employment as an emergency responder or any other reason deemed by the study team to be potentially related to their mental health; (6) were receiving another form of counseling for anxiety, depression, posttraumatic stress, or alcohol use problems. Attendance at self-help programs (e.g., Alcoholics Anonymous) and couples therapy was permitted, though no one in the sample reported attending a self-help program; or (7) endorsed drug use and symptoms consistent with a non-alcohol substance use disorder except nicotine, caffeine, or cannabis.

### 2.4 Procedure

The study took place between March 2022 and October 2023. Recruitment occurred via social media advertising and community presentations by the first author and staff affiliated with the Pitt Center for Emergency Responder Wellness. Potential participants emailed or called regarding their interest. A post-baccalaureate project coordinator provided study information and determined initial eligibility by phone using a structured screening questionnaire. This telephone screening included screening for the presence of a potential suicidal crisis. Any endorsement of thoughts of suicide in the past month was followed up with an immediate screening by a project clinician. No participants were excluded from the study based on presence of a suicidal crisis. Initially eligible participants were then sent the informed consent form electronically through REDCap. Informed consent and electronic data collection were completed using REDCap. Participants were given sufficient time to review it and were encouraged to reach out with any questions before providing their signature. Study staff then reached out to the participant to verbally confirm that the participant understood the procedures and to offer an additional opportunity to ask questions before the consent form was signed electronically and witnessed by study staff. Participants were sent a link to complete the baseline assessments online. Baseline questionnaires were reviewed to determine final eligibility. Individuals who were deemed ineligible to receive treatment through this project were referred to alternate, appropriate care. Eligible participants were then scheduled for the first treatment session. Participants met with therapists, who were assigned on a rotating basis, either in person at the university or via a secure, HIPAA-compliant online video conferencing platform, depending on the participant's preference. Treatment was delivered as individual (one-on-one) therapy. All treatment sessions were video recorded for training and quality control purposes. One month following the final treatment session, participants were emailed a link to complete the post-treatment assessments and, one month later, the follow-up assessments. Participants were sent reminders to complete these assessments via email as needed. Participants were compensated with a total of $120 for completing all assessments ($30 at baseline, $50 at post-treatment, and $40 at follow-up).

#### 2.4.1 Treatment

The UP (27, 28) is a transdiagnostic form of cognitive behavioral therapy that is empirically supported for treating a range of emotional disorders. In the UP, treatment mechanisms that underlie emotional disorders—NNE, difficulties in emotion regulation, and experiential avoidance—are targeted. These mechanisms are targeted via a series of treatment modules: (1) Assessment/Goal setting/Enhancing motivation, (2) Understanding emotions, (3) Mindful emotion awareness (1–2 sessions), (4) Cognitive flexibility (1–3 sessions), (5) Emotional behaviors (1–3 sessions), (6) Physical sensations (1–2 sessions), (7) Exposures (1–3 sessions), and (8). Recognizing accomplishments/Looking to the future. Standard delivery of the UP occurs across approximately 16 sessions. Treatment duration was tailored based on the individual needs of each participant. Eight sessions was considered the minimum threshold for treatment completion in this study. We arrived at this definition because it was as this was the minimum number of sessions needed to complete all 8 modules and is consistent with the definition of treatment completion used in our prior study of the UP in fire fighters (26). That said, there were instances in which a participant could be deemed a treatment completer by virtue of completing 8 or more sessions even if they did not complete all 8 modules, though this was rare.

#### 2.4.2 Cultural adaptation

Our prior study described in detail the cultural adaptations made to ensure the appropriateness of the UP content for use with fire fighter (26). During that prior study, our collaborators from fire service reviewed the UP materials for possible adaptations in the areas of language (Is terminology used in the UP appropriate and understandable for emergency responders?), concepts (Are concepts/modules likely to be relevant and helpful for emergency responders?), and specific examples (Are examples included in the UP materials likely to resonate? Are different examples needed?). Overall, our adaptation process highlighted the applicability of the standard UP with this population, along with the importance of attending to cultural considerations in certain instances. Specifically, we determined that no adaptations were needed pertaining to language or concepts. We made two adaptations regarding specific examples, both of which were flagged as potential “sticking points” that could interfere with treatment credibility when using the UP with emergency responders. First, we removed “avoiding caffeine” from the “Examples of Emotional Behaviors” worksheet in Module 5: Countering Emotional Behaviors. This example was deemed incongruent with norms among emergency responders, as caffeine intake is prevalent, particularly during overnight shifts. Second, we added an additional “thinking trap” to Module 4: Cognitive Flexibility. We added black-and-white/all-or-nothing thinking to characterize a range of potentially problematic thoughts commonly observed in emergency responders related to “worst case scenario” events to which first responders are likely to be exposed.

In the current study, we completed this same cultural adaptation process, this time with our study therapists from emergency medical service and law enforcement. Thus, across the two studies, the UP was reviewed for potential cultural adaptations by professionals from across fire, police, and emergency medical service who are also mental health professionals. The review during the current study led to one additional adaptation. Namely, given the prevalence of sleep problems in emergency responders, we decided that we would address maladaptive cognitions related to sleep problems (e.g., catastrophizing regarding sleep difficulties), as appropriate, as part of Module 4: Cognitive Flexibility.

#### 2.4.3 Clinicians, training, and supervision

Therapists included five masters-level students in clinical mental health counseling, one clinical social work masters student, one licensed clinical social worker (study author SR), and a licensed clinical psychologist (author EM). Therapist training included both UP-specific training activities as well as a multi-step process to ensure sufficient familiarity with emergency response culture. Study therapists read the UP Therapist Guide and Client Workbook (27, 28), attended a four hour training in using the UP with emergency responders led by a study author (EC), attended a two hour emergency responder cultural awareness training led by a study author (SR), and viewed previously completed videos of treatment sessions from this project. The two hour emergency responder cultural awareness training addressed topics including: training that emergency responders receive and how this could impact their responses to stress and trauma, the most challenging types of critical incidents to deal with emotionally, common reactions to trauma in emergency responders, compassion fatigue, impacts of emergency responder stress on family life, and the importance of relationship and social support for managing stress. Therapists completed a “ride-along” experiential training activity with a local emergency medical service. Throughout the study, the first author provided weekly supervision in groups of 2–3 clinicians during which review of segments of recordings of treatment sessions were reviewed and discussed on a rotating basis. The first author provided written feedback to clinicians on approximately 20% of treatment sessions. The study team benefited from including three therapists who are also emergency responders—two emergency medical technicians and one police officer. This, combined with the first author's experience with these occupational groups, enhanced our ability to focus on how emergency response cultural factors could be influencing the case conceptualization and treatment process during each of our supervision sessions.

### 2.5 Measures

#### 2.5.1 Eligibility screening measures

We screened for psychosis using the 6-item psychosis scale from the Psychiatric Diagnostic Screening Questionnaire [PDSQ; (75)]. We screened for violence risk using 4 items adapted from the Violence Risk Appraisal Guide (76). We screened for suicide risk using the suicide item from the Patient Health Questionnaire 9 Item- Depression [PHQ-9; (77)]. We used the 11-item Drug Use Disorders Identification Test [DUDIT; (78)] to screen for non-alcohol substance use disorder symptoms. We screened for potential presence of a suicidal crisis based on any endorsement of

#### 2.5.2 Sample characteristics

A self-report questionnaire was administered at baseline to assess demographic characteristics sex, race, age, marital status, education level, and emergency response service characteristics.

#### 2.5.3 Treatment outcome measures

##### 2.5.3.1 PTSD symptoms

The Posttraumatic Stress Disorder Checklist for DSM-5 [PCL-5; (79)] is a 20-item self-report measure of PTSD symptoms over the past month. It assesses the 20 DSM-5 symptom criteria for PTSD with responses rated on a 5-point Likert scale ranging from 0 to 4 (*0* *=* *not at all, 4* *=* *extremely*). The item scores are summed to creates a total symptom severity score ranging from 0 to 80. The PCL-5 has demonstrated good reliability and validity in emergency responders (26). We identified those who scored above a cutoff score of 41 on the PCL-5 as screening positive for PTSD at baseline (80). Clinically reliable change on this measure is 15–18, and an end-state score of ≤28 indicates clinically significant change (81). In the current study, Cronbach's alpha for the PCL-5 ranged from 0.94 to 0.96 across timepoints (pre-treatment, post-treatment, and one-month follow-up).

##### 2.5.3.2 Depression symptoms

The Patient Health Questionnaire 9 Item- Depression [PHQ-9; (77)] is a 9-item self-report measure that was used to screen for depression symptoms. The PHQ-9 asks patients to report how often- from 0- not at all to 3- nearly every day that they have been bothered by symptoms such as “little interest or pleasure in doing things,” and “feeling down, depressed or hopeless.” Items scores are summed to create a total score ranging from 0 to 27. Scores ranging from 0 to 4 indicate no depressive symptoms; 5–9 indicate mild depressive symptoms; 10–14 moderate; 15–19 moderately severe; 20–27 severe. In the current study, Cronbach's alpha for the PHQ-9 ranged from 0.87 to 0.89 across timepoints (pre-treatment, post-treatment, and 1-month follow-up).

##### 2.5.3.3 Anxiety symptoms

The GAD-7 (82) is a 7-item self-report measure that was used to screen for anxiety symptoms. The GAD-7 asks patients to report how often- from 0- not at all to 3- nearly every day- that they have been bothered by a series of problems such as “feeling nervous, anxious, or on edge,” and “worrying too much about different things.” Item scores are summed to yield a total score ranging from 0 to 21. Scores ranging from 0 to 4 indicate no anxiety symptoms; 5–9 indicate mild anxiety symptoms; 10–14 moderate; 15–21 severe. In the current study, Cronbach's alpha for the GAD-7 ranged from 0.93 to 0.94 across timepoints (pre-treatment, post-treatment, and one-month follow-up).

##### 2.5.3.4 Alcohol use disorder symptoms

The Alcohol Use Disorders Identification Test [AUDIT; (83)] is a widely used, 10-item measure of self-reported AUD symptoms and drinking quantity and frequency. Item response formats vary. Total scores of 10 or greater suggest probable AUD.

##### 2.5.3.5 Drug use disorder symptoms

The Drug Use Disorders Identification Test [DUDIT; (78, 84)] is an 11-item measure of self-reported non-alcohol substance use disorder symptoms that mirrors the AUDIT. Item response formats vary; total scores of 10 or greater suggest probable SUD.

##### 2.5.3.6 Sleep quality

The Pittsburgh Sleep Quality Index [PSQI; (85)] is a widely used, 18-item self-report measure that comprises seven component scores: (1) subjective sleep quality, (2) sleep latency, (3) sleep duration, (4) sleep efficiency, (5) sleep disturbances, (6) sleep medication usage, and (7) daytime dysfunction (85). Item formats vary. We examined the PSQI total score, with higher scores indicating worse overall sleep quality. For exploratory purposes, we also computed a score by summing a subset of PSQI items assessing aspects of sleep quality that may be more likely to change as a result of emotion-focused psychotherapy—these included subjective sleep quality, sleep latency, sleep efficiency, wakefulness after sleep onset, and use of sleep medications—as opposed to items more likely to be linked to sleep problems such as sleep apnea and therefore unlikely to improve via psychotherapy.

##### 2.5.3.7 Functional impairment

The Work and Social Adjustment Scale [WSAS; (86)] is a 5-item measure that was used to assess impairment in work, household tasks, social leisure activities, private recreation, and social relationships. Items are scored on a 0 (no impairment) to 8 (severe impairment) scale, with total scores ranging from 0 to 40. In the current study, Cronbach's alpha ranged from 0.86 to 0.89 across timepoints.

##### 2.5.3.8 Quality of life

The World Health Organization Quality of Life (87) is a 26-item self-report measure designed to assess participant satisfaction in four life domains: physical, psychological, social, and environmental. Items were scored on a 5-point Likert scale (*1* *=* *not at all, 5* *=* *extremely*). In this study, we used the total score. This measure has shown good reliability and validity in prior research with emergency responders (26). In the current study, Cronbach's alpha ranged from 0.92 to 0.93.

##### 2.5.3.9 Values-consistent actions

The Values Tracker [VT; (88)] is a two-item measure of the extent to which people report behaving in ways that are consistent with their personal values. Each item is rated on a 1–10 scale, with higher scored indicating greater values engagement.

#### 2.5.4 Treatment mechanisms

##### 2.5.4.1 NNE

The negative emotionality scale from the Big Five Inventory-2 (89) is a 12-item scale drawn from this larger personality inventory to assess NNE. In this study, reliability (Cronbach's alpha) ranged from 0.87 to 0.89 across time-points.

##### 2.5.4.2 Emotion regulation

The Difficulties in Emotion Regulation Scale-16 [DERS-16; (90);] is a 16-item self-report measure of frequency of difficulties in emotion regulation that is an abbreviated version of the lengthier DERS. It assesses four types of difficulties: non-acceptance of emotional responses, difficulty engaging in goal-directed behavior amidst emotional distress, difficulty with impulse control, and limited ability to engage in more helpful emotion regulation strategies. Items are scored on a scale of 1–5 with higher scores indicating greater more frequent difficulties in emotion regulation. Scores range from 16 to 80. In the current study, Cronbach's alpha ranged from 0.95 to 0.98.

##### 2.5.4.3 Experiential avoidance

The Acceptance and Action Questionnaire–II [AAQ-II; (63)] is a 7-item self-report measure of unwillingness or perceived inability to remain in contact with difficult internal experiences and efforts to escape, avoid, or modify these experiences. Items are scored on a scale of 1–7 with higher scores indicate greater experiential avoidance. In the current study, Cronbach's alpha ranged from 0.89 to 0.96.

##### 2.5.4.4 Therapeutic alliance

The Working Alliance Inventory—Short Form [WAI-SF; (91)] is a reliable and valid 12-item self-report measure of therapeutic alliance in three domains: agreement on therapy tasks, agreement on goals, and development of a therapist-client bond. Items are rated on a 5-point Likert scale ranging from 1 to 5 (*1* *=* *seldom, 5* *=* *always*). Item scores are summed to yield a total score ranging from 12 to 60, with higher scores reflecting better therapeutic alliance. The WAI-SF was administered at session 4. Internal consistency was 0.87.

### 2.6 Data analysis

We compared baseline demographic and clinical characteristics as a function of whether participants completed or dropped out of treatment using t-tests or Chi-square analyses, as appropriate. A series of paired-samples *t*-tests were conducted on all clinical variables comparing pre-treatment scores with scores on the outcomes at post-treatment and at one-month follow-up, along with repeated measures effect sizes (Hedges' *g*) where, by convention, values of .3, .5, and .8 are considered small, medium, and large, respectively. Next, because few studies have examined the UP in people with PTSD or AUD, we examined changes across outcomes in participants who screened positive for PTSD and AUD at baseline, respectively. Finally, we explored whether changes in the putative treatment mechanisms (WAI-SR, AAQ-II, DERS-16, BFI-2-N, PSQI) during treatment (i.e., pre- to post-treatment change scores) were associated with changes in the treatment outcomes during treatment and from pre-treatment to follow-up by computing Pearson product-moment correlations. Analyses were conducted using SPSS (v. 28).

## 3 Results

### 3.1 Enrollment and participant characteristics

A total of 39 people were screened for eligibility, of whom 36 were deemed eligible. Reasons for ineligibility included having a history of bipolar disorder (2) and not meeting our definition for being an emergency responder (1). Of these 36, 31 provided informed consent and completed the baseline assessment, whereas 5 decided not to participate prior to providing informed consent. One participant who provided informed consent did not complete the baseline assessment and was therefore ineligible. All 30 participants who completed the baseline assessment were deemed eligible and began treatment.

Sample characteristics for treatment completers and non-completers are described in Table 1. Participants were predominantly male (66.7%), Caucasian (93.3%), non-Hispanic (100%), with a mean age of 32.48 years (SD: 11.2 years, range: 19–57 years), 46.7% were single, 43.3% were married. On average, they had completed 14.89 years of education (SD: 1.7), 6.7% were military veterans, and the majority (73.3%) were emergency medical service providers (i.e., emergency medical technicians or paramedics). Emergency response telecommunicators (i.e., 911 dispatchers) were also eligible, though none enrolled in the study. Participants were offered the option of receiving care via video conference, in person, or via a combination of formats. The majority (*n* = 21; 70.0%) completed treatment via video conference, followed by a combination of formats (*n* = 5; 16.7%), and in-person only (*n* = 4; 13.3%). Among the 30 emergency responders who began treatment, 24 (80.0%) completed treatment, defined as attending 8 or more sessions. On average, treatment completers completed 15.41 sessions (range: 8–20), whereas those who dropped out completed an average of 6.17 sessions (range: 4–7). Clinical characteristics are also presented in Table 1. On average, participants scored above the recommended cutoff score for screening positive for PTSD among emergency responders on the PCL-5 (*M* = 43.10; *SD* = 19.2). Therapeutic working alliance, as measured by the WAI-SR, was high among treatment completers (*M* = 52.14 out of 60) as well as those who did not complete treatment (*M* = 50.0). Treatment completers did not differ from non-completers on any demographic, employment-related, or clinical characteristic. In terms of safety, there were two serious adverse events during the study (medical hospitalizations), neither of which were deemed by the IRB to be study related.

**Table 1:** Baseline demographic and clinical characteristics by treatment completion status.

Characteristics	Treatment completers (*n* = 24)	Non-completers (*n* = 6)	*p-*value
Age	32.74 (11.3)	31.50 (12.0)	.814
Years of education	15.00 (1.7)	14.50 (1.9)	.545
Sex: male; *n* (%)	16 (66.7)	4 (66.7)	.999
Ethnicity: Latinx *n* (%)	0 (0)	0 (0)	–
Race *n* (%)			.272
American Indian/Alaska Native	0 (0)	0 (0)	
Asian/Asian American	1 (4.1)	1 (16.7)	
Black or African American	0 (0)	0 (0)	
White	23 (95.8)	5 (83.3)	
Marital status *n* (%)			.332
Single	10 (41.6)	4 (66.7)	
Married or cohabitating	12 (50.0)	1 (16.7)	
Separated, divorced, or widowed	2 (8.3)	1 (16.7)	
Military veteran *n* (%)	2 (8.3)	0 (0)	.464
Type of emergency responder *n* (%)			.761
Emergency medical service (technician or paramedic)	17 (70.8)	5 (83.3)	
Fire fighter	2 (8.3)	0 (0)	
Law enforcement officer	3 (12.5)	1 (16.7)	
Emergency room medical provider	2 (8.3)	0 (0)	
Clinical/treatment characteristics			
PCL-5	43.25 (18.0)	42.50 (25.2)	.933
Positive screen for PTSD *n* (%)	11 (45.8)	4 (66.7)	.361
AUDIT	6.83 (7.2)	4.33 (5.8)	.437
Positive screen for alcohol use disorder *n* (%)	7 (29.2)	1 (16.7)	.536
PHQ-9	14.00 (6.0)	12.67 (9.5)	.667
GAD-7	11.83 (6.1)	14.17 (9.1)	.454
DUDIT	0.71 (2.0)	0 (0)	.397
Positive screen for drug use disorder *n* (%)	0 (0)	0 (0)	–
PSQI	11.33 (3.9)	8.67 (6.5)	.202
WSAS	17.63 (8.9)	19.17 (13.0)	.732
WHOQOL-total	85.92 (12.8)	84.33 (24.4)	.825
VT	11.92 (4.4)	10.50 (3.9)	.475
WAI-SR at session 4	52.14 (6.9)^a^	50.00 (7.3)^b^	.579
AAQ-II	28.96 (8.3)	28.83 (15.4)	.985
DERS	46.96 (15.2)	50.17 (20.4)	.668
BFI-2-N	38.13 (9.0)	40.50 (13.6)	.606

Data presented as *M* (*SD*) except where otherwise noted; PTSD, posttraumatic stress disorder. PCL-5, posttraumatic stress disorder checklist for DSM-5; PHQ-9, patient health questionnaire 9 Item- depression; GAD-7, generalized anxiety disorder-7; AUDIT, alcohol use disorders identification test; WSAS, work and social adjustment scale; WHOQOL, World Health Organization quality of life; VT, values tracker; PSQI, Pittsburgh sleep quality index; WAI-SR, working alliance inventory-self-report; AAQ-II, acceptance and action questionnaire-II; DERS, difficulties in emotion regulation scale; BFI-2-N, brief five factor inventory negative emotionality scale.

^a^ *n* = 21.

^b^ *n* = 4.

### 3.2 Main findings

Treatment outcome results are presented in Table 2. Of the 24 treatment completers, 20 (83.3%) completed a post-treatment assessment at the time of the final treatment session, and 15 (62.5%) completed the one-month follow-up assessment. There was a statistically significant reduction in self-reported PTSD symptoms on the PCL-5 at post-treatment, *p* < .001, *g* = 1.40 that remained significant at one-month follow-up, *p* = .002, *g* = 1.48 (large effects). The change in PCL-5 scores from pre-treatment to post-treatment (22.2 points) and follow-up (23.6 points) exceeded guidelines for determining clinically reliable change (81). There was a statistically significant reduction in depressive symptoms on the PHQ-9 at post-treatment that was sustained at follow-up, *p*-values <.001, *g*'s = 1.29 and 1.26, respectively (large effects). On average, scores were reduced from the moderate depressive symptoms range at pre-treatment to the mild symptom range at post-treatment and follow-up. There was a significant reduction in anxiety symptoms on the GAD-7 at post-treatment that was sustained at follow-up, *p*-values <.001, *g's* = 1.13 and 1.01, respectively (large effects). On average, scores were reduced from the moderate anxiety symptoms range at pre-treatment to the mild symptom range at post-treatment and the no symptoms range at follow-up. There was not a statistically significant reduction in symptoms of alcohol use disorder on the AUDIT at posttreatment or at follow-up (*g*'s = .22 and .23, negligible effects). There was a significant reduction in functional impairment on the WSAS at post-treatment that was sustained at follow-up, *p*-values <.001, *g's* = 1.16 and 1.07 (large effects). We observed a significant increase in overall quality of life on the WHOQOL at post-treatment that was sustained at follow-up, *p*-value =.007, *g's* = −.89 and −.81 (large effects). The increases in values-consistent actions on the VT were small at post-treatment (*p* = .333; *g* = −.34) and medium-to-large at follow-up (*p* = .050; *g* = −.77), though these changes were not statistically significantly different from zero. The improvement in sleep quality, assessed using the PSQI total scores during treatment (*p* = .071; *g* = .42, small effect) were not statistically significantly different from zero; however, PSQI total scores at follow-up were significantly greater than at pre-treatment (*g* = .69, medium effect, *p* = .008). On the subset of PSQI items thought to be more likely to improve among those receiving the UP, the effects were similar in magnitude compared to the PSQI total scores, though the change was statistically significant at both post-treatment (*p* = .023; *g* = .51, medium effect) and at follow-up (*g* = .57, medium effect, *p* = .011).

**Table 2:** Changes in treatment outcomes and treatment mechanisms between Pre-treatment and 1 month post-treatment in treatment completers.

Measure	Pre-treatment *n* = 24		Post-treatment *n* = 20			1 month post-treatment *n* = 15		
	M	SD	M	SD	g 95% CI	M	SD	g 95% CI
PCL-5	42.00	18.7	17.85	13.9	1.40*** .64–2.16	16.40	14.8	1.28** .41–2.16
PHQ-9	13.25	5.3	5.65	5.9	1.29*** .62–1.96	5.20	5.1	1.26*** .50–2.03
GAD-7	11.00	5.9	4.35	5.3	1.13*** .56–1.71	3.87	4.9	1.01*** .41–1.62
AUDIT	6.95	7.7	5.25	7.5	0.22 −.03 to .46	6.13	7.9	.23 −.05 to .51
WSAS	17.25	9.3	7.05	7.5	1.16*** .45–1.88	5.67	6.7	1.07** .22–1.92
WHOQOL-total	86.30	12.7	98.70	14.0	−.89** −1.46 to −.32	96.93	12.6	−.81** −1.45 to −.17
VT	12.35	4.5	13.90	4.3	−.34 −.99 to −.32	14.87	2.7	−.77 −1.59 to .06
PSQI	10.95	3.6	9.00	5.0	.42 −.06 to .90	8.67	3.2	.69** .14–1.24
AAQ-II	28.35	6.5	18.75	9.7	1.11** .40–1.82	17.27	9.6	1.23*** .42–2.03
DERS	44.45	13.4	30.35	15.2	.94*** .38–1.51	28.93	13.4	.99*** .35–1.63
BFI-2-N	38.13	9.0	26.29	11.8	1.09*** .46–1.72	29.00	9.1	.80** .20–1.39

PCL-5, posttraumatic stress disorder checklist for DSM-5; PHQ-9, patient health questionnaire 9 Item- depression; GAD-7, generalized anxiety disorder-7; AUDIT, alcohol use disorders identification test; WSAS, work and social adjustment scale; WHOQOL, World Health Organization quality of life; VT, values tracker; PSQI, Pittsburgh sleep quality index; AAQ-II, acceptance and action questionnaire-II; DERS, difficulties in emotion regulation scale; BFI-2-N, brief five factor inventory negative emotionality scale.

** *p* < .01; ***p < .001.

**Figure 1:**
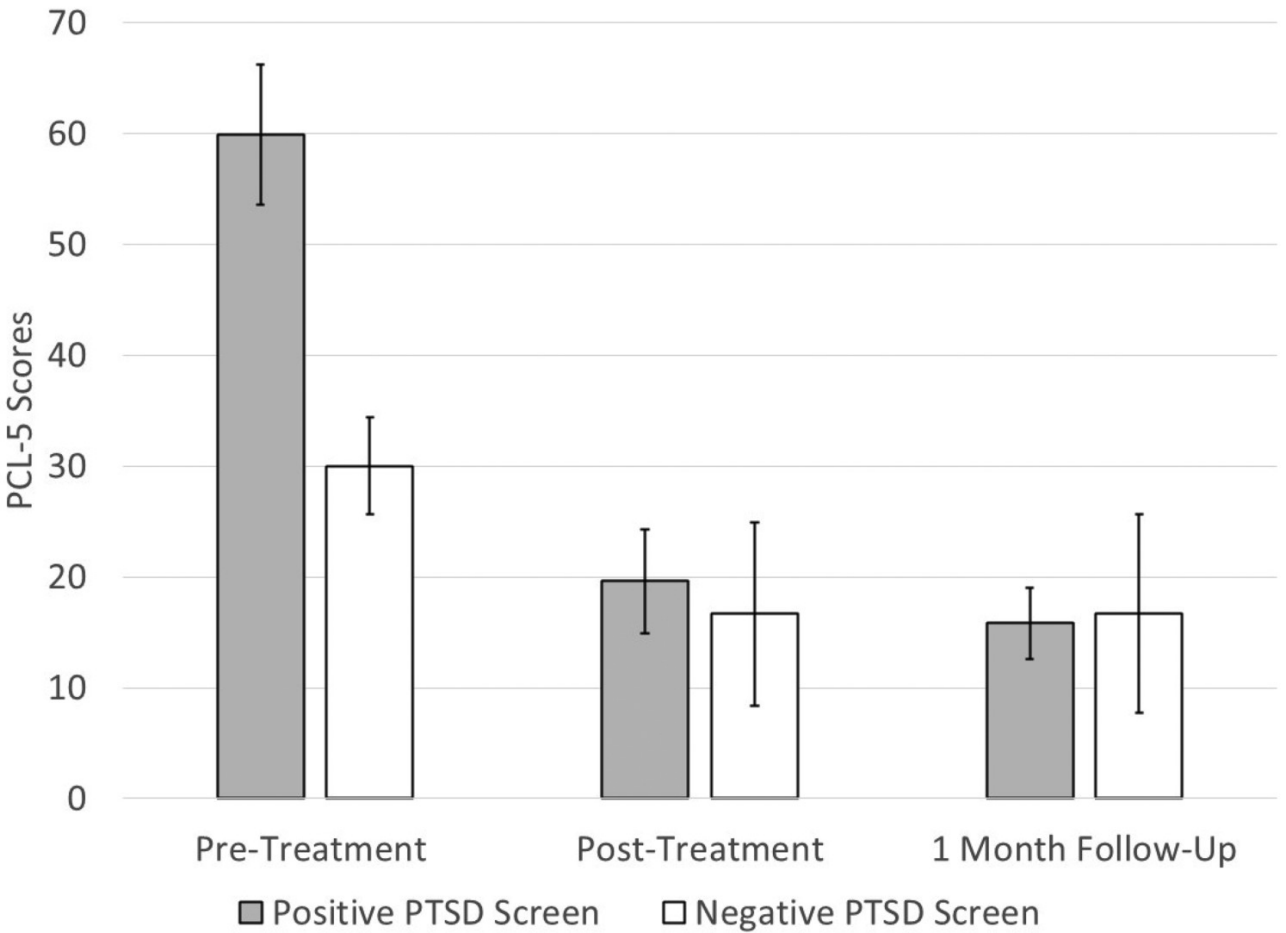
Changes in PCL-5 scores from pre-treatment to post-treatment and 1-month follow-up in those who screened positive and negative for posttraumatic stress disorder (PTSD) at pre-treatment*.* The error bars represent one standard deviation. The sample sizes are as follows: pre-treatment (*n* = 8), post-treatment (*n* = 8), follow-up (*n* = 6).

Next, we analyzed treatment outcomes for those who screened positive for PTSD at pre-treatment (Table 3). Half of the entire sample (*n* = 15; 50.0%) screened positive for PTSD at baseline. Nearly half of treatment completers (*n* = 11; 45.8%) screened positive for PTSD at pre-treatment, of whom 8 (72.7%) provided outcome data at post-treatment and 6 (54.5%) provided data at follow-up. Given the smaller sample, we focus on effect sizes in this summary of the findings, though results pertaining to statistical significance may be found in Table 3. In terms of the overall pattern of findings, the effect sizes at post-treatment and follow-up were larger for those who screened positive for PTSD at pre-treatment compared with the total completers sample on most outcome measures. There were large reductions in PTSD symptoms on the PCL-5 at post-treatment (*g* = 2.92) and follow-up (*g* = 3.12). The changes in PCL-5 scores at post-treatment (40.3 points) and follow-up (44.1 points) exceeded guidelines for clinically reliable change. As presented in Figure 1, those who screened positive for PTSD at pre-treatment reported symptom severity equivalent to those who had screened negative for PTSD at both post-treatment and follow-up. There were large reductions in depressive symptoms on the PHQ-9 at post-treatment and follow-up (*g* = 2.29 and 2.49, respectively). On average, scores were reduced from the moderately severe depressive symptoms range at pre-treatment to the mild symptom range at post-treatment and follow-up. There was a large reduction in anxiety symptoms on the GAD-7 at post-treatment (*g* = 1.39) that was reduced to a medium effect at follow-up (*g* = .61). On average, scores were reduced from the moderate anxiety symptoms range at pre-treatment to the no symptom range at post-treatment and follow-up. There was no change in symptoms of alcohol use disorder on the AUDIT at posttreatment or follow-up (*g*'s = .10 and.14, negligible effects). We observed large improvements in functional impairment on the WSAS at post-treatment and follow-up (*g* = 2.17 and 2.57). We observed large increases in quality of life on the WHOQOL at post-treatment and follow-up (*g* = −.93 and −1.61) and on values-consistent actions on the VT at post-treatment and follow-up (*g* = −.88 and −1.81). There was a small-to-medium sized improvement in PSQI total scores at post-treatment (*g* = .46) and a large effect at follow-up (*g* = 1.89). On the subset of PSQI items thought to be more likely to improve among those receiving the UP, the effects were similar compared to the PSQI total scores at post-treatment (*g* = .60, medium effect) and at follow-up (*g* = 1.02, large effect).

**Table 3:** Changes in treatment outcomes and treatment mechanisms between pre-treatment and 1 month post-treatment in participants who screened positive for posttraumatic stress disorder at baseline.

Measure	Pre-treatment *n* = 8		Post-treatment *n* = 8			1 month post-treatment *n* = 6		
	M	SD	M	SD	g 95% CI	M	SD	g 95% CI
PCL-5	59.88	14.6	19.63	9.8	2.92** .66–5.19	15.80	6.4	3.12** −.33 to 6.56
PHQ-9	16.13	4.7	5.38	3.5	2.29** .49–4.09	5.40	2.2	2.49* −.45 to 5.42
GAD-7	14.00	6.9	4.75	4.4	1.39** .16–2.61	3.40	1.8	.61* −.23 to 1.46
AUDIT	6.88	11.1	5.50	9.9	.10 −.07 to .28	8.20	11.5	.14 −.16 to .45
WSAS	22.13	6.3	7.25	5.9	2.17** .37–3.96	5.20	4.1	2.57* −.50 to 5.65
WHOQOL-Total	80.75	10.7	95.75	16.6	−.93* −1.95. to .09	93.00	11.9	−1.61* −3.82 to .59
VT	11.38	5.2	15.25	2.6	−.88 −2.36. to .60	14.60	2.5	−1.81 −4.38 to .77
PSQI	12.25	2.6	10.38	4.5	.46 −.77 to 1.68	9.6	2.3	1.89* −.30 to 4.08
AAQ-II	33.63	5.0	19.50	7.9	1.92** .22–3.62	17.60	6.7	2.29** −.16 to 4.74
DERS	52.25	6.8	30.38	11.8	1.99** .41–3.57	28.20	10.1	1.94** −.14 to 4.03
BFI-2-N	42.91	8.7	26.00	13.6	1.38** .21–2.55	29.40	6.8	1.14 −.61 to 2.89

PCL-5, posttraumatic stress disorder checklist for DSM-5; PHQ-9, patient health questionnaire 9 Item- depression; GAD-7, generalized anxiety disorder-7; AUDIT, alcohol use disorders identification test; WSAS, work and social adjustment scale; WHOQOL, World Health Organization quality of life; VT, values tracker; PSQI, Pittsburgh sleep quality index; AAQ-II, acceptance and action questionnaire-II; DERS, difficulties in emotion regulation scale; BFI-2-N, brief five factor inventory negative emotionality scale.

* *p* < .05. **p < .01.

Within the total sample, 8 (26.7%) screened positive for AUD. Among treatment completers, 7 (29.2%) screened positive for AUD, of whom 6 completed the post-treatment assessment and 5 provided follow-up data. AUDIT scores decreased from *M* = 15.3 (*SD* = 8.8) at pre-treatment to *M* = 11.5 (*SD* = 11.2) at post-treatment (*g* = .32; small effect) and from *M* = 16.4 (*SD* = 9.5) at pre-treatment to *M* = 12.6 (*SD* = 10.9) at follow-up (*g* = .30; small effect).

### 3.3 Treatment mechanisms

Changes in the putative treatment mechanisms are presented in Tables 2, 3. We observed large, statistically significant changes in each of the putative treatment mechanisms (psychological inflexibility on the AAQ-II, difficulties in emotion regulation on the DERS, and NNE on the BFI-2-N) during treatment and at follow-up in the total sample of treatment completers. We observed a similar pattern of large, statistically significant changes in each of the treatment mechanisms at follow-up, with the exception that the change in scores on the BFI-2-N was not statistically significantly different from zero at follow-up despite a large effect (*g* = 1.14) due to the small sample size.

We explored the associations between changes in the treatment outcomes during treatment and at follow-up and changes in the treatment mechanisms during treatment. These results are presented in Table 4. Correlations between reductions in each of the mechanistic variables and improvements in mental health symptoms (PCL-5, PHQ-9, GAD-7) were uniformly high, with *r*'s ranging from .533 to .866 (*p*-values .016 to <.001). Correlations between improvements in AUD symptoms on the AUDIT and reductions in the treatment mechanisms during treatment were not significant. At follow-up, improvements in AUDIT at follow-up were associated with reductions in AAQ-II (*r* = .613, *p* = .015) and DERS (*r* = .528, *p* = .043). There were large associations between improvements in sleep quality on the PSQI and reductions in all three mechanistic variables at post-treatment and at follow-up (*r's* .549 to .690; *p*-values .012–.004), with the exception that the correlation with reduction in BFI-2-N was not statistically significant at follow-up (*r* = .472, *p* = .076). There were large associations between improvements in functional impairment on the WSAS and reductions in all three mechanistic variables at post-treatment and at follow-up (*r's*. 711 to .889; *p*-values.001 to <.001), with the exception that the correlation with reduction in BFI-2-N did not reach statistical significance at post-treatment (*r* = .408, *p* = .074). Improvements in quality of life on the WHOQOL were associated with reductions in all three mechanistic variables at post-treatment (*r's* .588 to. 741; *p*-values.006 to <.001), whereas none of these correlations were significant at follow-up (*r*'s .217 to .393). At post-treatment, increased engagement in values-consistent behaviors on the VT was associated with reductions in AAQ-II (*r* = .450, *p* = .046) and DERS (*r* = .540, *p* = .014), whereas the association with changes in BFI-2-N was not statistically significant (*r* = .442, *p* = .051). At follow-up, changes in VT scores were not associated with changes in any of the mechanistic variables. For comparison, the association between therapeutic alliance on the WAI-SR and changes in treatment outcomes were much smaller and were not statistically significantly different from zero for any treatment outcome. Overall, there was little difference in the mean correlation between changes in these mechanistic variables and changes across all outcomes: AAQ-II mean *r* = .60; DERS = .58; BFI-2-*N* = .49.

**Table 4:** Correlations between changes in treatment mechanisms during treatment and changes in treatment outcomes at post-treatment and at follow-up and between therapeutic alliance and changes in treatment outcomes.

Change in outcome	WAI-SR^a^	AAQ-II	DERS	BFI-2-N	PSQI
PCL-5 pre-post	.117	.858***	.789***	.594**	.588**
PCL-5 pre-follow-up	.098	.821***	.866***	.687**	.724**
PHQ-9 pre-post	.200	.665**	.539*	.533*	.541*
PHQ-9 pre-follow-up	.338	.711**	.590*	.666**	.574*
GAD-7 pre-post	.064	.655**	.579**	.547*	.576**
GAD-7 pre-follow-up	.115	.665**	.650**	.717**	.675**
AUDIT pre-post	-.015	.336	.350	.165	.272
AUDIT pre-follow-up	.143	.613*	.528*	.368	.279
WSAS pre-post	.101	.711***	.718***	.408	.467*
WSAS pre-follow-up	.164	.889***	.778***	.752**	.790***
WHOQOL total pre-post	.336	.686***	.588**	.741***	.600**
WHOQOL total pre-Follow-up	.049	.244	.217	.393	.121
VT pre-post	.162	.450*	.540*	.442	.609**
VT pre- follow-up	-.276	-.033	.257	-.267	.476
PSQI pre-post	.152	.636**	.576**	.549*	–
PSQI pre-follow-up	.035	.690**	.659**	.472	–

PCL-5, posttraumatic stress disorder checklist for DSM-5; PHQ-9, patient health questionnaire 9 Item- depression; GAD-7, generalized anxiety disorder-7; AUDIT, alcohol use disorders identification test; WSAS, work and social adjustment scale; WHOQOL, World Health Organization quality of life; VT, values tracker; PSQI, Pittsburgh sleep quality index; WAI-SR, working alliance inventory-self-report; AAQ-II, acceptance and action questionnaire-II; DERS, difficulties in emotion regulation scale; BFI-2-N, brief five factor inventory negative emotionality scale.

* *p* < .05. **p < .01. ***p < .001.

^a^ WAI-SR was administered following session 4.

Finally, because improvements in sleep quality may drive improvements in mental health symptoms and related outcomes, we examined the association between changes in PSQI total scores and changes in the other treatment outcomes (Table 4). Improvements in PSQI were associated with reductions in mental health symptoms at post-treatment and at follow-up (*r's*.541 to.724; *p*-values .014–.002). Improvements in PSQI were not associated with reductions in AUDIT scores at post-treatment or follow-up. Improvements in PSQI were associated with improvements in WSAS at post-treatment and follow-up (*r*'s.467 and.790, *p*-values.038 to <.001, respectively). Improvements in PSQI were associated with improvements in WHOQOL at post-treatment (*r* = .600, *p* = .005) but not at follow-up. Improvements in PSQI were associated with improvements in VT at post-treatment (*r* = .609, *p* = .004), though this correlation did not reach statistical significance at follow-up (*r* = .476, *p* = .073).

## 4 Discussion

Emergency responders are essential public safety workers that experience elevated rates of a number of emotional disorders linked to frequent trauma exposure, shift work and other factors that interfere with quality sleep, and myriad other occupational stressors. Multiple barriers impede treatment seeking in this population including lack of provider knowledge regarding emergency response culture, concerns about receiving treatment for symptoms linked to occupational stress, and scheduling challenges. The UP is an evidence-based approach to addressing inter-connected symptoms of emotional disorders; yet there are few published data on the UP in trauma exposed populations. One prior study examined the UP, culturally adapted for fire fighters, and delivered via video conferencing. That study reported strong feasibility, acceptability, and improvements in mental health symptoms and quality of life in fire fighters (26). The current study built on that prior study by examining a version of the UP that was more broadly culturally adapted for use across emergency response professions. In the current study, the UP was delivered, either in person or via video conferencing, to a mixed sample of emergency responders predominantly comprised of emergency medical professionals.

In terms of treatment feasibility and acceptability, we observed an (80.0%) treatment completion rate, which is comparable to that observed in our prior study of the UP delivered exclusively via video conferencing in fire fighters (80.3%). These treatment completion rates are high for a trauma exposed population (92, 93). Participants in this study reported that their working alliance with their clinician was quite strong, replicating our prior findings regarding therapeutic alliance. In the current study, the majority of treatment (70.0%) was delivered via video conferencing or via a combination of in-person and video (17.3%), indicating that this has become a preferred format within this population. Convenience was cited most often as the reason for this, though alleviation of concerns about stigma and privacy may also have been a factor.

All emergency responders are exposed to potentially traumatic events, and half of the current sample met screening criteria for PTSD. Among treatment completers, we observed large, statistically significant and clinically meaningful reductions in PTSD symptom severity that was maintained at one-month follow-up. These effect sizes closely mirrored—and were slightly larger than—the effects observed in the prior study of the UP in fire fighters. Similarly, we observed large, statistically and clinically significant effect size improvements in symptoms of depression and anxiety at post-treatment that were maintained at follow-up. Again, the magnitude of these improvements closely mirrored but was somewhat larger than the effects observed in the study of the UP with fire fighters. Reductions in symptoms of PTSD, depression, and anxiety were even greater among the subgroup of participants who screened positive for PTSD at pre-treatment. By post-treatment, these participants reported PTSD symptom severity levels that were lower than the pre-treatment PTSD symptom levels for those who screened negative for PTSD. Among participants who screened positive for AUD, we observed small reductions in AUD symptoms. This finding differs from our prior study (26) in which we observed large improvements in AUD symptoms. Of note, the sample size was much smaller in the current study (*n* = 6), which also used a different measure of AUD than our prior study. Additional research is warranted regarding the effect of the UP on symptoms of AUD in trauma exposed populations.

We also observed strong links between the UP and improvements in several other outcomes. We observed large effect size improvements in quality of life at post-treatment and follow-up, which replicated the finding from the prior study of fire fighters. We also observed large effect size improvements in a measure of impairment in occupational and social functioning at post-treatment and follow-up. We administered a measure of engagement in behaviors that participants view as being consistent with their sense of personal values, meaning, and purpose. On this measure, we observed small effect size improvements at post-treatment and medium-to-large effects at follow-up. This finding, while moderate in magnitude, is noteworthy given that engagement in values-consistent action is not an explicit goal within the UP as in other transdiagnostic treatments such as Acceptance and Commitment Therapy. Next, we examined changes in sleep quality, which is understudied within the broader context of mental health treatment in emergency responders. Moreover, this is a novel area of inquiry, with no published RCTs of the UP for sleep problems to date. At post-treatment, we observed small effect size improvements in sleep quality. However, by follow-up, these improvements represented a medium effect and were statistically significant. Although our adaptation of the UP included greater emphasis on sleep problems than is present in the standard UP, additional modifications may be warranted given the high prevalence of sleep problems in emergency responders. For example, incorporating stimulus control techniques and providing psychoeducation regarding shift work and sleep may confer additional benefits. Next, reductions in alcohol misuse symptoms were small and were not statistically significant. The effect of the UP on alcohol misuse symptoms was somewhat larger in the prior study of the UP in fire fighters in which the proportion of participants who screened positive for AUD was greater than in the current study. The mixed findings across these two studies indicate that the UP has potential for reducing alcohol misuse, though future work should focus on tailoring skills within the UP toward reducing reliance on alcohol as an emotion regulation strategy within the context of co-occurring mental health challenges. Examining whether the UP is associated with reductions in AUD symptoms is also novel, as no published trials have examined this. Overall, these findings, particularly when combined with the prior study in fire fighters, indicates that the UP is a strong match for efficiently addressing the behavioral health needs of emergency responders living with the most common types of challenges observed in this occupational group.

We sought to validate and enhance understanding of treatment mechanisms targeted by the UP, particularly within a trauma exposed sample given the lack of data in this population. We observed large, statistically significant changes in each of these mechanistic variables—NNE, difficulties in emotion regulation, and experiential avoidance—at both post-treatment and follow-up. We observed large associations between reductions in each of these variables during treatment and improvements in mental health symptoms at post-treatment and at follow-up (mean *r* = .67). Reductions in functional impairment were associated with reductions in each of the mechanistic variables (mean *r* = .77), with the exception of a medium-sized correlation with reductions in NNE at post-treatment. Changes in each of the mechanistic variables were linked with improved quality of life at post-treatment, though not at follow-up. Interestingly, improved engagement in values-consistent behavior was moderately linked with changes in each of the mechanisms at post-treatment, though not at follow-up. Whereas experiential avoidance has been discussed in the literature as impeding consistent values engagement, these findings suggest that high levels of negative emotion may be more broadly linked with this outcome. We observed similarly large links between changes in these mechanistic variables and improved sleep quality across both measurements (mean *r* = .60). This finding provides evidence that changes in emotion-related treatment mechanisms are linked with improved sleep and underscores the potential of emotion-focused treatment such as the UP for addressing sleep problems in the context of co-occurring mental health symptoms. Finally, there is no clear consensus in the literature regarding whether it makes sense to target sleep problems as a prelude to addressing trauma related mental health symptoms vs. addressing trauma related symptoms first and then treating any residual sleep problems following treatment for PTSD [see (94) for discussion of this issue]. Therefore, we examined changes in sleep quality during treatment as a mechanistic variable. Improved sleep quality during treatment was associated with most outcome variables at post-treatment and follow-up, indicating that improved sleep quality may help to explain changes—or maintenance of changes—in mental health symptoms and functional outcomes in people receiving the UP.

This study was novel in piloting a culturally adapted version of the UP, which has not yet been extensively tested as a treatment in trauma exposed samples, with emergency responders, an understudied group in relation to the size of this population and the frequency of trauma exposure that they encounter. In terms of clinical implications, our cultural adaptation process, which now includes review by fire, police, and emergency medical personnel across two studies, indicates that only a small number of modifications to the UP were warranted when using the UP with emergency responders. Our adaptations were minor; they consisted of (1) removing a suggestion related to avoiding caffeine because of norms around caffeine use among emergency responders, (2) adding all-or-nothing thinking as a thinking trap to which emergency responders are frequently susceptible, and (3) probing regarding the presence of sleep-related thinking traps given the prevalence of sleep problems in emergency responders. Thus, those who are trained in using the UP but who have little experience working with emergency responders may find that adding these adaptations, along with receiving basic training in emergency response culture, may be sufficient to support them in working with this population.

Strengths of the current study include use of well-validated, widely used self-report measures and broad eligibility criteria, both of which are likely representative of procedures used in clinical practice. The findings of this study should be interpreted in the context of several limitations. The lack of a control group precludes our ability to infer causality regarding the impact of the UP on changes in the outcomes. The racial and ethnic diversity within this sample was low, though it was likely broadly representative of the demographics within emergency response departments—particularly emergency medical services and fire service—in our region. Results pertaining to the subgroup of participants who screened positive for PTSD should be interpreted with particular caution, owing to the small sample size, though these results were consistent with those observed in a subsample that screened positive for PTSD in a prior study of the UP in fire fighters that had a somewhat larger sample (26). Our sample largely consisted of emergency medical service personnel, whereas we had anticipated having a more mixed sample of emergency responders. We received many word-of-mouth referrals to the study within emergency service departments, underscoring the need for services and satisfaction with the treatment received through this project. The generalizability of these findings to other emergency responder professions may be limited, though we again reference the similarity of the current findings to our prior study of fire fighters (26). While we believe that the cultural adaptations enhanced the impact of the UP with this population, our study design did not allow us to empirically evaluate the impact of these adaptations. The small sample in this pilot study precluded examining treatment mechanisms using mediation analyses, which would be preferred for examining these relationships. A larger sample would also allow for more complex modeling analyses that would allow for testing of multiple mediators simultaneously to determine which variables are influencing changes in the outcome while accounting for the other mediators. Finally, although the study therapists received training and ongoing supervision in delivering the UP, the lack of formal fidelity monitoring is a limitation. Overall, the current findings support use of the UP and add to the burgeoning literature on the UP in trauma exposed populations, including emergency responders. The current findings indicate that controlled research is warranted examining the UP in trauma exposed populations. Future, controlled research with a larger, more diverse sample would allow for conclusions to be drawn regarding treatment efficacy, more rigorous examination of treatment mechanisms, and more generalizable findings.

## Data Availability

The raw data supporting the conclusions of this article will be made available by the authors, without undue reservation, pending approval of a data use agreement.
